# Past, Present, and Future Strategies for Enhanced Assessment of Embryo's Genome and Reproductive Competence in Women of Advanced Reproductive Age

**DOI:** 10.3389/fendo.2019.00154

**Published:** 2019-03-19

**Authors:** Maurizio Poli, Laura Girardi, Marco Fabiani, Martina Moretto, Valeria Romanelli, Cristina Patassini, Daniela Zuccarello, Antonio Capalbo

**Affiliations:** ^1^IGENOMIX, Marostica, Italy; ^2^REPROOMICS, Amsterdam, Netherlands; ^3^Clinical Genetics Unit, University Hospital of Padova, Padua, Italy; ^4^IGENOMIX, Parque Tecnologico Paterna, Valencia, Spain; ^5^Sezione Istologia ed Embriologia Medica, Dipartimento di Scienze Anatomiche, Istologiche, Medico-Legali e dell'Apparato Locomotore, University of Rome “La Sapienza”, Rome, Italy

**Keywords:** PGT, embryo (human), diagnostics, *in vitro* fertilization (IVF), genetics

## Abstract

Recent advancements in genomic analysis allow testing of an increasing number of genetic features in human preimplantation embryos. Typical single gene mutation and whole chromosomes testing can now be integrated with assessment of mitochondrial DNA and polygenic conditions. Diagnostic expansion into epigenetic and transcriptomic assessment in the near future are potential technological targets which may improve the prognostic outlook of patients of advanced reproductive age and overall *in vitro* fertilization (IVF) treatment outcomes. In this review, we discuss the technological progress of recent years and their future applications in preimplantation genetic testing in IVF.

## Introduction

**Figure 1:**
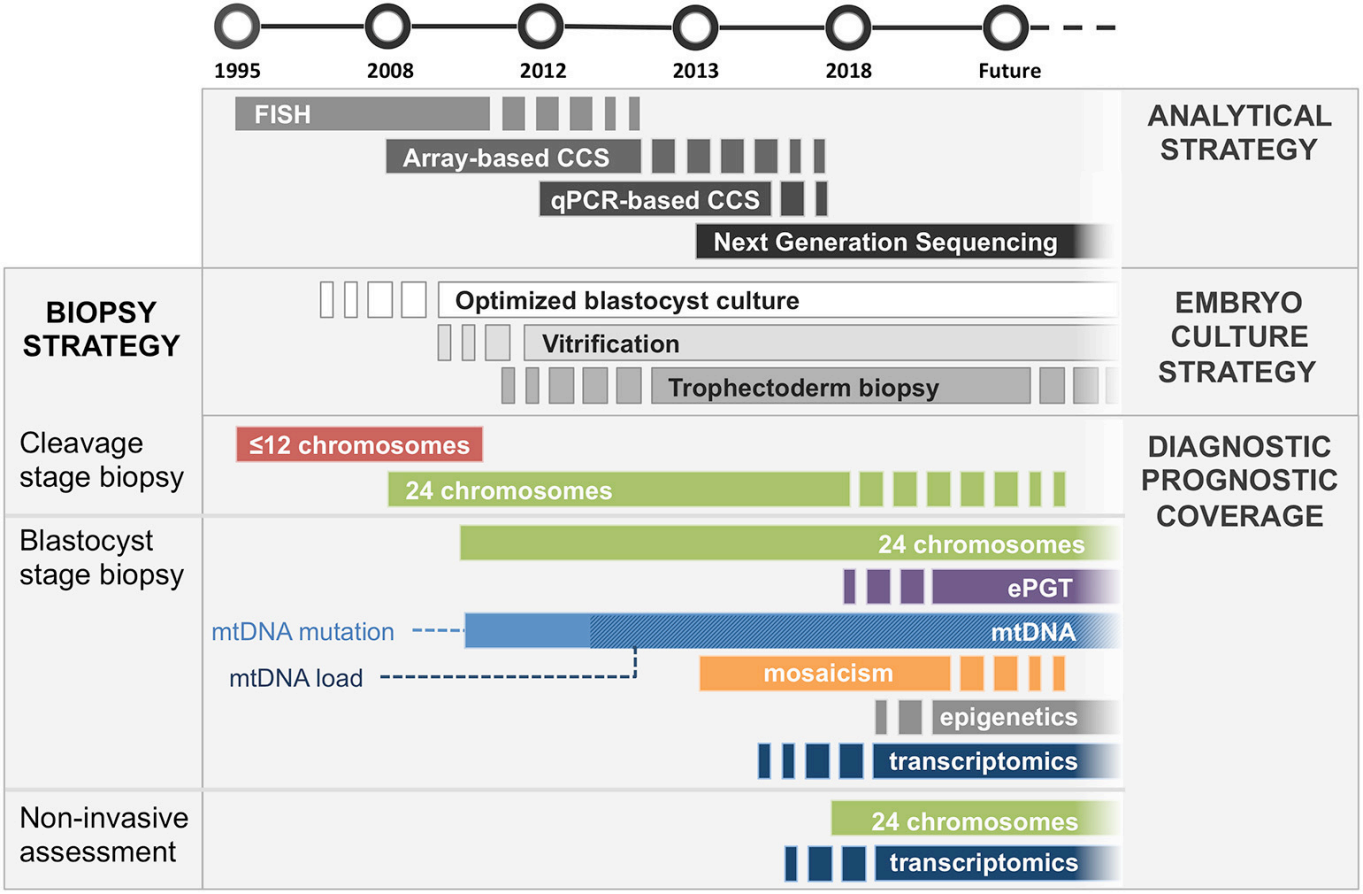
Timeline of introduction to clinical practice of embryological and analytical achievements. *Analytical strategy, dark gray bars section*: FISH was first employed for assessment of a limited amount of chromosomes in mid ‘90s, followed by microarray-based comprehensive chromosomal screening techniques (e.g., aSNP, aCGH) in mid 2000's. Comprehensive quantitative PCR methods were introduced in ‘10s, shortly followed by NGS-based methods, which is now employed for most of chromosomal screening analyses, progressively replacing other less sensitive, more expensive and labor intensive techniques. *Embryo culture strategy, light gray bars section:* Blastocyst culture is still being introduced into clinical settings (ongoing since 2000's). Similarly, embryo vitrification is increasingly being adopted since its introduction in mid 2000's. Trophectoderm biopsy is currently the gold standard for embryo genetic assessment; however, it may become replaced by non-invasive approaches for certain applications (i.e., PGT-A) should they demonstrate high sensitivity and specificity in large clinical trials. *Diagnostic/prognostic coverage, colored bars section: Cleavage stage biopsy:* FISH was first employed in cleavage stage embryos. It was later completely replaced by comprehensive CCS strategies, which enabled analysis of all chromosomes. Cleavage stage biopsy is still employed by IVF laboratories not equipped for blastocyst culture. *Blastocyst stage biopsy:* Trophectoderm cells provide the most robust specimen to generate genetic analysis on. Therefore, this strategy is applicable to all diagnostic approaches including embryo karyotyping, expanded mutation screening, mitochondrial DNA analysis and mosaicism detection, as well as future additional clinically employable methodologies analyzing epigenetic and trascriptomic features of the embryo. *Non-invasive assessment:* Genetic analysis of spent culture media provides the safest and least invasive approach. Clinical application of this strategy would allow assessment of the chromosomal complement of embryonic cells as well as the analysis of embryo-derived transcripts with paracrine/autocrine effect (i.e., miRNA). Its suitability for analysis of factors with typical intracellular localization (i.e., mitochondria, epigenetic features) is still being investigated. It is unlikely that niPGT will be able to provide meaningful data on mosaicism status of the embryo. Nonetheless, this application requires further data to reach an exhaustive answer.

Preimplantation genetic testing (PGT) is a methodology designed to assess the genetic complement of embryos generated during *in vitro* fertilization (IVF) treatments. Its current applications include both the detection of monogenic disorders and chromosome copy number. Over the years the molecular strategies employed for PGT have changed and improved, taking advantage of the technological progressions introduced in molecular genetics (Figure 1, Table 1). In parallel, improvements in culture systems and cryopreservation protocols employed in IVF allowed the production of more robust results from genetic testing and additional analytical flexibility and opportunity, enabling simultaneous evaluation of reproductive features of IVF embryos beyond genetic and chromosomal status (Figure 1, Table 1).

**Table 1:** Summary of technical characteristics, advantages and disadvantages of methodologies employed in current and future PGT applications.

		**Diagnostic employment**	**Technology characteristics**	**Advantages**	**Disadvantages**
Biopsy strategy	Polar bodies	Indirect assessment of oocyte's chromosomal content	a) Double biopsy of individual PBs on Day 0 and Day 1	Analysis performed on waste products of female meiosis	Analysis limited to the maternal genome (excludes paternal genome and potential mitotic errors)
			OR	Allowed in countries where embryo biopsy is banned	Biopsy and analysis of both PBs required (time-consuming and non-cost-effective procedure)
			b) Single biopsy of both PBs on Day 1	Compatible with fresh ET after genetic diagnosis	Technical issues associated to single cell-based analysis
					High amplification failure rate (≈10%)
	Blastomere	Direct assessment of embryo's chromosomal status	Single or double blastomere biopsy on Day 3	Vast experience worldwide	High impact on embryo reproductive competence
				Embryos reach the cleavage stage in a synchronous fashion (day 3 of preimplantation development)	Technical issues associated with single cell-based analysis
				Standardized approach	Lowest diagnostic reliability
				Compatible with fresh ET	High amplification failure rate (≈10%)
	Trophectoderm	Direct assessment of embryo's chromosomal status	5–8 cell trophectoderm biopsy on Day 5, 6, or 7, depending on embryo developmental rate	Robust diagnosis (5–10 cells are retrieved and analyzed)	The IVF laboratory must be experienced in extended embryo culture and vitrification
				Selection of TE cells to biopsy. The ICM is not involved	Limited experience worldwide
				Highly cost-effective (biopsy of developmentally-competent embryos only)	Most commonly incompatible with fresh embryo transfer
				No impact on embryo reproductive competence	
				Low amplification failure rate (≈1%)	
PGT-A	FISH	Assessment of the copy number of a limited set of chromosomes	Fluorescent probes for specific chromosomes are hybridized with single cell DNA. Number of chromosomes is inferred from the number of probe's signals	First genetic methodology employed for chromosomal assessment	Allows screening of some of the chromosomes only
					Labor intense
					Incompatible with multicellular biopsy (TE)
	aCGH	Comprehensive assessment of the copy number of all chromosomes	DNA content extracted from a specimen is amplified and labeled with a fluorochrome and cohybridised with a normal reference DNA labeled with a different color onto a microarray slide carrying DNA fragments representative of a whole karyotype. Color ratios identified through HD scans refer to different chromosomal statuses	Compatible with both multicellular biopsy and single cell analysis Comprehensive testing of all 24 chromosomes	Low sensitivity for mosaicism and segmental aneuploidies Expensive and labor intense
	qPCR	Comprehensive assessment of the copy number of all chromosomes	Unique sequences in each chromosome are selectively amplified using RealTime PCR. Amplification curves for each chromosome are compared across them and with a reference. Steeper curves correlate to higher amounts of starting material (trisomy), slower curves with fewer starting material (monosomy).	Compatible with both multicellular biopsy and single cell analysis Comprehensive testing of all 24 chromosomes Cheap and automatable Compatible with combined PGT-A and PGT-M analysis	Low sensitivity for segmental aneuploidies
	NGS	Comprehensive assessment of the copy number of all chromosomes	DNA content extracted from biopsy specimen is amplified, fragmented and tagged with sample-specific sequences. Multiple samples can be parallely sequenced using different technologies. Sequences generated are searched on genomic database to identify their location on the genome. Comparison between number of reads representing specific regions are used to infer chromosomal copy number.	Compatible with both multicellular biopsy and single cell analysis Comprehensive testing of all 24 chromosomes High sensitivity for segmental aneuploidies and mosaicism Increasingly cheap and automatable Compatible with combined PGT-A and PGT-M analysis	
	Nanopore	Comprehensive assessment of the copy number of all chromosomes	Lysated cells are loaded on a Nanopore DNA sequencer where an electrical current feeds single strand DNA through a flow-cell membrane whilst recording voltage changes occurring in the protein nanopores. This information is then translated into DNA sequences that are subsequently aligned to a reference DNA and analyzed for chromosomal copy number variation.	Compatible with both multicellular biopsy and single cell analysis Comprehensive testing of all 24 chromosomes Cheap cost of analytical unit Reduce overall time for analysis and allow fresh embryo transfer also in blastocyst biopsy cases Small device footprint. Potential to be installed within the IVF laboratory (technical personnel and diagnostic accreditation still required)	Unknown sensitivity for mosaicism and segmental aneuploidies Low base calling precision Currently not applicable for PGT-M purposes
PGT-M	Direct + Linkage analysis	Direct and indirect detection of presence of single gene mutation	Custom primers are employed to amplify the specific genetic region containing the mutation to investigate. Product amplification are subjected to mini-sequencing to determine the allelic status.	Robust diagnostic strategy	Requires custom made probes for each couple tested
				Possible to detect *de novo* mutations	Test set-up and validation is time consuming
				Applicable to almost all cases	
				Low implementation costs	
	Karyomapping	Indirect assessment of presence of single gene mutation through haplotyping	DNA content extracted from a biopsy specimen is amplified and labeled with a fluorochrome and hybridized onto a microarray slide carrying 300,000 SNP variants. Embryo's haplotype is reconstructed based on the frequencies of informative SNPs.	Applicable with minimal patient-specific custom set up	Not applicable if affected gene is located in a region with insufficient SNP markers
				Short work up time	Requires DNA analysis of an affected person in the family to set up the test
					Inapplicable in cases involving *de novo* mutation
					High costs of implementation
	NGS	Direct assessment of presence of single gene mutation	Targeted amplification of region of interest followed by sequencing and reads analysis	Multi-gene analysis	High analytical costs (current)
Additional markers for embryo selection	ePGT-M	Parallel assessment of Mendelian and multi-factorial genetic inheritance	Based on SNP array and bioinformatic algorithms.	Simultaneous assessment of pathogenetic and predisposing conditions	Ethical considerations to be expertly evaluated
	mtDNA mutation	Assessment of mutation load in mitochondrial genome	Custom primers are employed to amplify the specific mtDNA region containing the mutation to investigate. Product amplification are subjected to genotyping to determine mutation status.	Diagnosis of pathogenic mitochondrial conditions	Difficult interpretation of results due to heteroplasmy
	mtDNA load	Assessment of amount of mitochondria present in biopsied specimen	Highly conserved mtDNA regions are targeted in Real Time PCR amplification. Amplification curves are compared with internal standards	Additional data on cellular energetic supply and metabolism for embryo selection purposes	Unproven in large RCT studies
	Epigenetics	Assessment of inheritable and induced epigenetic alterations	Alternative approaches possible	Additional data on embryo viability status for selection purposes	
	Transcriptomics	Monitoring/assessment of developmental processes ongoing in the embryo	Alternative approaches possible	Additional data on embryo viability status for selection purposes	
niPGT-A		Non-invasive assessment of embryo's chromosomal status	Cell-free DNA is amplified and subjected to NGS protocol	Embryonic cells are not removed for diagnostic purposes	Diagnostic accuracy and sensitivity must be proven in large clinical studies
				Potential universal applicability	

In this article, we review the molecular and embryological strategies employed so far, including future technological developments for increasing the scope of genetic assessment and complementary non-invasive approaches especially focused on counteracting the genetic risks associated with advanced reproductive age.

## Biopsy Strategy

Embryo biopsy represents a crucial step in embryo genetic assessment both from biological and technical standpoints. Originally, embryo biopsy was performed on cleavage stage embryos by removing one (or two) cell(s) from an 8-cell embryo. Over the years, once enough data was collected, this practice has shown several limitations (1). Most importantly, the removal of one cell from cleavage stage embryo was shown to have an impact on its developmental and reproductive potential due to the risk of removing cells already committed to inner cell mass lineage differentiation (2). The series of molecular events ultimately leading to symmetry breaking and the timing and progression of cell lineage commitment are poorly characterized in human embryos. Additionally, the homogeneity in appearance of cleavage stage blastomeres hinders any possibility to evaluate embryo's cell lineage commitment progression morphologically, thus leaving to chance the risk of removing an ICM-committed cell during biopsy.

The development of extended culture systems has allowed the postponement of embryo biopsy to the blastocyst stage, where embryo genome activation is complete and cell lineage commitment becomes morphologically detectable. Additionally, by collecting 5–8 cells from the trophectoderm (TE) wall, the proportion of the embryonic biomass removed at the blastocyst stage is lower compared to cleavage stage (3–5 vs. 12%, respectively) and selectively obtained from the extra-embryonic lineage. The intrinsic advantages of blastocyst biopsy, combined with the development of comprehensive chromosome screening platforms (CCS), led to the development of a more robust diagnostic strategy. Firstly, the higher number of cells collected provides a larger amount of DNA template for downstream amplification and analysis. This feature reduces significantly the proportion of failed amplification and allele drop out events, increasing technical reproducibility and diagnostic rates. Secondly, multiple cell analysis generates more robust results compared to single cell analysis, minimizing the risk of false positive and false negative results associated with single cell analysis. Lastly, TE biopsy followed by vitrification yields to higher survival rates compared to slow freezing of biopsied cleavage stage embryos (3). The higher results are due to the lower water content of TE cells compared to blastomeres, the reduced risk of intracellular ice crystal formation associated with vitrification and the higher structural flexibility of blastocysts compared to cleavage stage embryos (4). All these factors have contributed to the development of a global strategy for the investigation of embryos genetic features, based on multicellular samples supplying reliable source of template DNA for robust downstream analysis.

Finally, from a clinical viewpoint, the exclusive analysis of developmentally competent embryos resulted in a more cost-effective diagnostic strategy compared to previous approaches employing PBs or blastomeres, leading to a rapid expansion of PGT practice to more clinics and patients over the last years. Indeed, a recent survey from the ESHRE PGD consortium showed an increasing trend toward blastocyst biopsy in recent years and that in 2016 it was the main strategy for preimplantation genetic testing of embryos (roughly 60% of all PGT procedures, data from ESHRE PGD consortium at the 2018 Annual Meeting in Barcelona).

Patients in advanced reproductive age opting for PGT analysis benefit from blastocyst biopsy strategy in three main ways. Through extended culture they (a) allow the assessment of their embryos' ability to develop passed the cleavage stage (Day 3), (b) reduce extra analytical costs by limiting the number of embryos to test, and (c) receive robust diagnostic results upon which meaningful clinical decisions can be made.

## Chromosomes

Chromosome analysis has been employed clinically since the mid ‘90s (5, 6). This type of analysis is performed to ensure that the embryo transferred to the patient has a correct number of chromosomes, thus reducing the risks of abnormal pregnancy, miscarriage, and failed implantation. Initially, this diagnostic strategy was performed by fluorescent *in situ* hybridization (FISH), involving the spreading of a single cell biopsied from a cleavage stage embryo on a glass slide and the hybridization of its DNA with chromosome-specific fluorescent probes. Several limitations of this approach have been described in the literature, including the limited amount of chromosomes that could be simultaneously assessed and the high incidence of false positive results (7, 8). The limited number of chromosome probes evaluated also meant that some aneuploidies were left untested, resulting in undetected aneuploid embryos being transferred. In the mid 2000's, it became clear that these shortcomings and diagnostic unreliability, coupled with the negative consequences of cleavage stage biopsy, were compromising clinical outcomes of patients undergoing PGT, highlighting the necessity of a safer, more robust and precise strategy (9). Subsequently, the development of comprehensive chromosome screening technologies (CCS), including comparative genomic hybridization arrays (aCGH) and single nucleotide polymorphisms arrays (SNP arrays) and quantitative polymerase chain reaction (qPCR), provided significant improvement to PGT clinical application. These technologies not only are able to accurately evaluate all 24 chromosomes in a single analysis, but also are applicable to single cells with sufficient accuracy. When tested on single cell from fibroblast cell lines with known karyotype, all platforms provided accuracy rates above 98% for whole chromosome aneuploidies (10–14). The largest comparative study between two methodologies (aCGH and qPCR) conducted on embryo biopsies reported high concordance across the two platforms (15). In this study, qPCR and aCGH showed similar sensitivity (98.2 vs. 98.8%, respectively, not significant), whereas qPCR displayed a significantly higher specificity compared with aCGH (99.9 vs. 99.6%, respectively, *P* = 0.01) (15). Despite the need for larger comparative studies, technological performance appears to be similar across all platforms when standard criteria for diagnosis of whole chromosome aneuploidies are used.

All CCS strategies allow parallel sample analysis and produce higher throughput compared to FISH. Also, some of the CCS strategies now available avoid time consuming and high labor-intensive steps required for FISH analysis, allowing more reproducible and streamlined processing conditions (i.e., qPCR, aCGH). In recent years, Next Generation Sequencing (NGS) platforms have been adapted for embryo aneuploidy testing using low-depth genome sequencing and copy number variation analysis. Due to its sensitivity, coupled with further extended chromosome coverage, NGS provides higher accuracy in the assessment of sub-chromosomal abnormalities (i.e., segmental aneuploidies) compared to previous CCS methods (16). Additionally, NGS is currently employed for the detection of chromosomal mosaicism, where two karyotypically different cell populations coexist in the same embryo. Despite lacking significant level of diagnostic validation, NGS was suggested to be able to detect low-level mosaicism (i.e., 20%) and accurately discriminate the proportion of cells showing abnormal karyotype (17). Nonetheless, mosaicism detection at low and high levels (e.g., 20 and 80%, respectively) is yet to be confirmed as a true biological finding, rather than a technical variation, hence its clinical impact still requires evidence support (18–20). This point is of extreme importance, especially for patients producing a limited amount of embryos per cycle, like advanced maternal age patients. Indeed, no embryos should be wasted due to diagnostic uncertainties, even more so in those cases where patients are unlikely to generate more embryos. Today, the main advantage provided by NGS in PGT is the possibility to analyze multiple samples in parallel, thus bringing a significant reduction of both costs and sample running time and allowing a wider accessibility to PGT for patients worldwide. Similarly to other qPCR platforms, NGS is compatible with combined assessment of both aneuploidy and single gene mutation, where the initial round of whole genome amplification (WGA) is integrated with targeted amplification of loci of interest (21–23).

The latest technology appearing in the preimplantation genetics landscape is Nanopore sequencing. This sequencing strategy is completely different from NGS as it relies on voltage changes occurring in protein nanopores fixed across a flow-cell membrane. Pre-amplified single-stranded DNA molecules are driven through the nanopores by an electrical current, whilst variable resistivity produced by the passage of each nucleic base is recorded (24). This information is then translated into DNA sequences that are subsequently aligned to a reference DNA and analyzed for chromosomal copy number variation. A small cohort study has recently shown that this technology is able to identify whole chromosome abnormalities in blastocyst biopsies with a sensitivity equal to NGS (25). Nanopore technology presents several advantages including a significantly lower price per analytical unit (~$1,000 for a MinION vs. >$99,000 for a MiSeq), a smaller footprint in the laboratory, and the possibility of in-house genetic analysis for IVF laboratories, thus reducing overall time for analysis (~2 h) and allowing for fresh embryo transfer after PGT. On the other hand, Nanopore strategy doesn't allow detection of mosaicism or segmental aneuploidies unless deeper sequencing experiments are performed. Additionally, Nanopore single-base calling sensitivity and precision requires validation to determine its suitability for more hi-depth sequencing tasks. Moreover, its implementation in IVF laboratories would still require technical expertise, dedicated clean room facilities for pre-amplification steps and WGA, as well as professional accreditation to carry out molecular biology diagnostics.

Current technology allows PGT laboratories to handle high volumes of samples for CCS tests, providing a very valuable tool for couples of advanced reproductive age undergoing IVF.

IVF patients of advanced maternal age mainly benefit from PGT by avoiding the transfer of chromosomally impaired embryos. In these patients, the likelihood of producing abnormal embryos is significantly higher than in younger populations, hence aneuploidy testing is often recommended. Although preimplantation genetic testing for aneuploidies (PGT-A) doesn't improve cumulative clinical pregnancy rates per cycle started (as it can't improve or repair chromosomal abnormalities), it reduces the chance of miscarriage and failed implantation, thus increasing overall pregnancy rates per transfer and minimizing time to pregnancy (26, 27).

Nonetheless, some controversies still surround the application of PGT-A, especially in relation to the clinical impact of mosaicism detection and segmental aneuploidies. Several groups are currently investigating these topics in an effort to clarify the real incidence of mosaicism in human embryos, whilst identifying a clinical management strategy that minimizes both embryo wastage and risks to patients.

Additionally, the clinical implementation of biopsy procedures can be challenging for smaller clinics both from a financial and logistic standpoint due to the purchase of equipment required for PGT (i.e., laser) and operators training.

## Single Gene Mutations and Expanded Preimplantation Genetic Testing

Prior to aneuploidy testing, preimplantation genetic testing was mainly employed for the detection of inheritable genetic conditions in embryos generated by couples with increased reproductive risk (28). This risk is determined by the presence of a pathological mutation in the genome of one or both the prospective parents. The main diagnostic strategy employed for single gene mutation detection has been only slightly changed from the first cases. Preimplantation genetic testing for mutations (PGT-M) involves the direct analysis of the region of the mutation performed by targeted amplification of the small region of the genome including the mutation site, followed by genetic variant detection by mini-sequencing or qPCR based genotyping. This analysis is strengthened by parallel multiplex amplification of informative highly polymorphic markers flanking the mutation site in order to counterbalance the risk of allele drop out at the mutation site and minimize the chance of no diagnosis. Similar to genetic testing for aneuploidy, the limiting factor in single gene disorder assessment is the amount of DNA available in single diploid cells, as a successful diagnosis is dependent on the detection of both alleles (maternal and paternal), each present in a single copy. Blastocyst biopsy is also beneficial in this type of analysis, increasing the starting template DNA material and thus reducing the risk of either DNA amplification failure (AF), target DNA contamination and allele dropout (ADO), in which one of the two alleles is preferentially amplified to the detriment of the other.

Karyomapping is a more recent diagnostic strategy for PGT-M that takes advantage of the presence of almost 300,000 SNPs scattered throughout the genome, which provide linkage analysis capabilities for virtually every region of interest. Using the data previously generated from both parents and an affected family member DNA samples, it is possible to reconstruct the segregation patterns of the mutation and assess its presence in the embryo. This indirect linkage analysis allows the detection of most pathological variants using a global platform that doesn't require patient-specific custom set-up. Despite its broad applicability, Karyomapping shows diagnostic limitations in cases where insufficient informative SNP markers are found in the region of interest (e.g., telomeric genes). Furthermore, the application of Karyomapping requires the analysis of at least one affected family member in addition to the two parents during set-up. If this reference sample is unavailable (around 30–40% of cases), a classic approach must be employed. Also, Karyomapping cannot be applied in cases involving *de novo* mutations, or when there are cases of recombination that make family samples non-informative (29), falling short of providing a universal approach for PGT-M analysis.

More recently, the development of NGS platforms has provided the possibility to combine the assessment of single gene mutations and aneuploidies on the same biopsy. Additionally, an alternative NGS-based approach aimed at the amplification of customizable genomic loci has been investigated (t-NGS) (21). This methodology involves a multiplex PCR that amplifies sequences necessary to assess both aneuploidy status and pathological mutations of a gene of interest, without providing data on the entire genome (21, 30, 31). However, due to the high sequencing depth required for accurate single nucleotide call in single cell genomics, NGS-based methods for PGT-M are still far from being cost-effective compared to previously established procedures and are therefore not commonly employed in current clinical practice. In the future, a reduction of sequencing costs, combined with improved automation, is expected, likely making NGS-based PGT-M feasible in a clinical context.

Nonetheless, by using combined approaches, it is possible to assess both the presence of single gene disorders and karyotype of the embryo from the same biopsy specimen (23, 32). This approach is especially recommended for patients of advanced maternal age carrying an inheritable genetic condition. In these cases, the embryo is at high risk not only of inheriting the genetic mutation, but also a chromosomal abnormality derived from age-related defective meiosis. Consequently, the cohort from whom the embryo is selected for transfer is dramatically reduced. For this reason, the treating clinician should carefully estimate patients' ovarian reserve and overall reproductive potential in order to provide them with adequate counsel.

The application of genome-wide technologies to PGT is also paving the way for screening of multifactorial conditions, where the assessment of multiple genes and epigenetic factors will enable clinical risk calculations for the onset of diseases not following Mendelian inheritance. This further evaluation of embryo's polygenic and multifactorial conditions might represent a possible solution for IVF patients, which may enable additional parameters for embryo ranking based on lower risk factors. Genome-wide SNP-array and NGS technologies have the potential to be employed clinically for the detection of relevant alleles included in polygenic risk scores panels (33). This approach, called “extended preimplantation genetic testing” (ePGT) can be employed to screen embryos for further genetic features, additional to known, full-penetrance conditions (autosomic dominant and recessive) running in the family (34). These include both incomplete penetrance conditions (i.e., cancer predisposition - *BRCA1* and *BRCA2* genes), but also multifactorial/polygenic conditions (i.e., congenital diabetes, cardiomyopathies, hypertension, hypothyroidism). This type of enhanced genetic assessment would allow a more thorough analysis of embryo's genetic inheritance, further restricting the total genetic risk passed on to the offspring, not only including mutations running in the family but also more common multifactorial conditions with strong genetic bases and *de novo* conditions and mutations exclusively present in the germinal line. With the evolution and full integration of genetics into clinical management of potentially every pathological and sub-pathological condition, expanded preimplantation genetic testing (ePGT) would enable the application of early interventions for medically actionable conditions in the prenatal or perinatal stages, providing tailored and efficient treatment of various conditions and improving overall prognosis and lifestyle of future generations.

Nonetheless, although providing additional testing capabilities and diagnostic coverage of pathological statuses, expanded genetic assessment would also involve potential incidental findings that may affect the life of the prospective parents, especially regarding late onset and incomplete penetrance conditions. Also, the actual prognostic predictive ability of several newly developed polygenic risk scores derived from population studies are yet to be validated and their potential clinical implementation should be further assessed and investigated. For this reason, the ethical consequences and the management of diagnostic results derived from expanded genetic testing of preimplantation embryos should be carefully evaluated for each individual case prior to its clinical implementation (35).

Ethical issues surrounding the application of ePGT and its preconceptional version ePCS (expanded preconception carrier screening, carried out on prospective parents rather than embryos) involve patient's well-being from a legal and psychosocial standpoint. Indeed, ePGT and ePCS should be offered to the general public only when its effects on voluntary informed consensus is granted by the patient. Additionally, the impact of ePGT/ePCS on individuals' psychological health and social fairness should be carefully investigated in order to warrant overall safety of the analysis. This assessment should include the impact of test results on psychological well-being, patient's perception of health and related false reassurance, as well as potential stigmatization, discrimination, legal inequity and unfairness derived from unfavorable results (36).

Although technical limitations and ethical concerns will require resolving prior to its clinical application, it is likely that ePGT will become applicable to the general IVF population in order to prevent the transmission of a wide spectrum of inheritable genetic conditions to their offspring.

## Non-mendelian Genetic Factors

Parallel to nuclear genome analysis, additional genetic features are critical for both the transmission of inheritable conditions (i.e., mitochondrial disorders) and embryo developmental and implantation competence. Despite its ability to identify embryos with no reproductive potential (e.g., aneuploid embryos), PGT-A has limitations in identifying euploid embryos capable of implantation and further development. Indeed, only around 50% of euploid embryos transferred to patients go on to implant (37). Moreover, around 10% of the euploid embryos that implant end up miscarrying prior to 12 weeks of gestation, suggesting the need for more comprehensive testing approaches to assess extra molecular markers for reproductive competence. Additional embryo genetic features, including mitochondrial genome, epigenetic background and transcriptional activity could be analyzed to further assess their relation with embryo's reproductive capabilities.

## Mitochondrial DNA

### Mitochondrial Diseases

Mitochondrial disorders are characterized by their exclusive maternal inheritance. The presence of pathological mutations in the mitochondrial genome (mtDNA) is a phenomenon due to both inheritance and spontaneous *de novo* occurrence due to the lack of DNA error checking mechanisms in the mitochondrion (which are instead present in the nucleus). Each mitochondrion contains multiple molecules of mtDNA, each of them potentially differing from the others (heteroplasmy). Each mitochondrion replicates autonomously within the cell, reaching a total number of hundreds of organelles. Mitochondrial pathological symptoms arise when the number of defective organelles passes the threshold of expression. Most of these pathological conditions involve energy production processes carried out in the mitochondrion, ultimately affecting several organs, especially targeting muscle and nervous tissues (i.e., myopathy, encephalopathy, neuropathy). For instance, mitochondrial disorders include Leber's hereditary optic neuropathy (LHON) where a point mutation occurs in one of the genes coding for protein subunits forming the NADH dehydrogenase enzyme, affecting the activity of oxidative phosphorylation complex I located in the mitochondrion inner membrane (nucleotide positions 11778 G to A, 3460 G to A and 14484 T to C, respectively in the ND4, ND1 and ND6 subunits) and eventually causing optic atrophy. A rare variant of this condition named LHON Plus also involves the loss of brain ability to control muscular activity including the cardiac muscle.

Due to the random nature of mitochondrial replication and consequent heteroplasmy ratio, mutated mtDNA load might require careful assessment prior to embryo transfer. Additionally, due to the high mutation rate in mtDNA and the complex penetrance mechanism, every person is at risk of harboring a potentially life-threatening mitochondrial condition.

For this reason, following technological and clinical validation, the integration of mitochondrial testing in conventional PGT would further expand the coverage of embryo genetic assessment, ensuring the identification of nuclear and mitochondrial mutation-free embryos. Similarly to cases where nuclear single gene mutations are investigated, aneuploidy assessment should also be performed for advanced maternal age. In order to produce realistic reproductive estimations, ovarian reserve should also be tested prior to commencing IVF/PGT-A/PGT-mitochondria treatment for these patients. Nonetheless, representativeness of biopsied cells of the mitochondrial constitution of the whole embryo still requires further investigation and extensive validation.

### Mitochondrial Load as Embryo Viability Marker

The number of mtDNA molecules present in a biopsy has been proposed as a marker for embryo reproductive competence (38, 39). It has been shown that significantly high concentration of mtDNA is associated with aneuploidy and lower implantation abilities. Concentration thresholds were proposed, however these parameters showed low sensitivity in defining embryo implantation potential (40). Additionally, confirmatory studies from other groups have failed to validate the initial findings and others have shown completely inversed outcomes (41, 42). Nonetheless, the identification of molecular markers of embryo viability is of crucial importance for the field and, should initial findings be corroborated by additional evidence in future studies, mtDNA may be used for this purpose.

## Epigenetic Landscape

Preimplantation embryos undergo major chromatin restructuring in the period between fertilization and implantation. Due to technical limitations associated with single cell genomics, these mechanisms and the extent of these processes have been ignored in humans until recent times. Animal models were first investigated, however, direct evidence of similar epigenetic strategies on humans have been lacking. Recent studies have shown the possibility of investigating chromatin rearrangements and gene accessibility in the early developmental stages following fertilization (43–45). Using single-cell chromatin overall omic-scale landscape sequencing (scCOOL-seq), Li et al. were able to simultaneously analyze several molecular epigenetic characteristics of embryonic genome including chromatin state, nucleosome positioning, DNA methylation and the interrelationship among different epigenetic layers in the same individual human preimplantation embryonic cells (44). This study provided a temporal landscape of the epigenetic status changes throughout development from gamete to blastocyst, revealing insights on DNA accessibility and transcription rates at different preimplantation stages. Another group investigating IVF embryos undergoing TE biopsy performed DNA sequencing after bisulfite conversion and used the methylation data to infer both transcription rates and chromosome copy number variation (46). According to this study, methylation levels of low-quality blastocysts diverge from those of high-quality blastocysts. Additionally, authors suggest that the presence of abnormally methylated regions is associated with the failure of embryonic development and live birth (46). This type of approach may be used in the future not only to assess embryo's chromosome composition but also to add prognostic outlook on its developmental and reproductive potential. Should oocyte aging be shown to impact epigenetic remodeling of maternal and embryonic genomes, the evaluation of these markers in embryo biopsies may potentially be used to further improve embryo selection between euploid embryos, especially in patients of advanced maternal age.

## Transcriptomics Assessment

The impact of both infertility and IVF treatment on embryo development is poorly understood and although IVF is generally considered to be safe, it has been suggested that artificial conditions may have an impact on cell lineage differentiation processes and epigenetic conformation of embryo's genome that may result in fetal developmental and neonatal complications. Using an murine model, Giritharan and colleagues showed that ICM and TE of *in vitro* developed blastocysts showed lower transcriptional differences compared to *in vivo* developed blastocysts, suggesting a compromised cell differentiation mechanism (47). Additionally, a more recent study on human blastocysts showed that embryo transcriptional profiles are affected by the type of media used for culture, highlighting the impact of culture conditions on embryo development and the necessity of increase the understanding of this process (48). A recent study comparing RNA-Seq generated transcriptional maps of human blastocysts derived from young and older patients highlighted a significant age-dependent reduction in transcriptional activity for more than 800 genes (49). Biological functions of down-regulated genes included “cell cycle control” and “metaphase checkpoint regulation,” suggesting their concurrent role in the instauration of suboptimal chromosomal segregation processes in advanced maternal age patients. The translation of these results into a diagnostic approach improving reproductive outcomes for these patients is currently under evaluation.

Although these findings require further confirmation and validation, transcriptomic profiles may be targeted in future PGT assessment strategies to identify embryos with normal developmental competence and improve embryo selection.

## NON-invasive PGT

Currently, trophectoderm biopsy provides the most robust and reliable source of embryonic DNA for the analysis of embryo's genetic features. However, despite having been shown that TE biopsy doesn't affect embryo's reproductive potential (2), there is increasing interest in reducing or completely avoid intervention on the embryo for diagnostic purposes (50). Non-invasive assessment not only would completely avoid any potential injury to the embryo, but it would also predispose the genetic analysis to full automation and operator-independence. Approaches alternative to embryo biopsy that have been investigated thus far include both minimally invasive aspiration of the blastocoel fluid (BF) [e.g., Blastocentesis, (51–53)] and fully non-invasive analysis of spent culture media (54, 55). Although initial tests of both these strategies have shown reduced overall diagnostic power (suboptimal DNA amplification rates, reduced concordance rate with TE results, inconsistence of results across groups) (56), the high potential benefits brought by their clinical application warrant further investigation. For example, the main setbacks from culture media testing have been reported to be the low DNA amplification rate and the high detection of contaminating DNA of maternal origin (56). These problems may be successfully resolved in the future by tweaking embryo culture conditions and sample collection. For instance, smaller volumes of media may be used to allow for greater DNA concentration in the culture drop, whilst serial embryo washes prior to specimen collection may ensure the complete disposal of maternal DNA from persisting granulosa cells or degenerating polar bodies. With the aim of an unmanned, uncontaminated and standardized global strategy for molecular analysis of spent culture media, microfluidics technology involving automated media changeover and collection may also be developed and implemented in future incubators. This will allow the establishment of a consistent, reproducible and comprehensive approach to embryo's genome evaluation and embryo selection employable in all IVF treatments without additional workload on the embryology laboratory.

## Conclusions

Recent genomics technological achievements and optimization of embryo culture systems have created a robust PGT methodology, on the base of which solid data and reliable diagnostic conclusions can be generated. This strong analytical platform has indeed enabled the implementation of chromosomes and single gene disorders testing in an increasing portion of IVF treatment cycles, improving clinical outcomes for specific patients populations (i.e., patients at increased risk of generating genetic or chromosomally abnormal embryos). Benefits of chromosomal assessment include the increase in embryo implantation per transfer and an overall reduction in miscarriage rates. These aspects are even more crucial for advanced maternal age patients where majority of embryos are impaired by age-dependent chromosomal abnormalities. Nonetheless, current PGT approaches have margin for improvement, especially for what it concerns parallel chromosomal and single gene testing. Additionally, to ensure that the embryo transferred is free of life-impairing mutations, the mutations tested should not be limited to a specific inheritable one running in the family, but extended into a more comprehensive panel of common conditions, including analysis of *de novo* mutations occurring in the germline and more common genes/variants related with multifactorial inheritance in the context of ePGT. In the future, supplementary details regarding embryo's reproductive competence and its ability to implant and generate a healthy pregnancy may be acquired by the analysis of additional epigenetic parameters and transcription profiles which might be as well-integrated in the PGT workflow. These complimentary analyses have the potential to further improve clinical outcomes for patients across all ages. However, they may be extensively applied for advanced maternal age patients where success rates are time-sensitive and an accurate evaluation of embryonic development and implantation competence is paramount not only for embryo selection purposes, but also for decisions related to patients future treatment options and chances of success. Although unlikely to be compatible with epigenetic assessment, non-invasive approaches are being investigated for both single gene and chromosomal testing, as well as for discovering alternative markers for embryo paracrine signaling and reproductive competence. Non-invasive approaches represent an extremely interesting field of development for PGT as it would pave the way for its widespread application and full integration into the IVF treatment.

Nonetheless, similarly to other novel genetic testing strategies, the technical ability to perform molecular analyses has occurred at a faster rate than the evidence required for optimal integration could be comprehensively collected and validated. For this reason, it will be crucial for all novel applications discussed in this review to be fully tested, optimized and monitored before they can be offered to patients in a clinical context.

## Author Contributions

MP, LG, MF, MM, VR, CP, DZ, and AC drafted the manuscript. MP and AC revised the manuscript.

### Conflict of Interest Statement

MP, LG, MF, VR, CP, and AC are employed by iGenomix Italy. MP is also employed by Reproomics BV. The remaining authors declare that the research was conducted in the absence of any commercial or financial relationships that could be construed as a potential conflict of interest.
